# Editorial: Natural products based management of neurological disorders: Mechanistic insight and translational informatics approach

**DOI:** 10.3389/fphar.2023.1170839

**Published:** 2023-03-07

**Authors:** Suresh Kumar, Sevgi Gezici, Nazim Sekeroglu, Atanas G. Atanasov, Rajeev K. Singla

**Affiliations:** ^1^ Department of Pharmaceutical Sciences and Drug Research, Punjabi University, Patiala, Punjab, India; ^2^ Faculty of Medicine, University of Gaziantep, Gaziantep, Türkiye; ^3^ Department of Biology, Faculty of Arts and Sciences, University of Gaziantep, Gaziantep, Türkiye; ^4^ Ludwig Boltzmann Institute Digital Health and Patient Safety, Medical University of Vienna, Vienna, Austria; ^5^ Institute of Genetics and Animal Biotechnology of the Polish Academy of Sciences, Magdalenka, Poland; ^6^ Institutes for Systems Genetics, Frontiers Science Center for Disease-related Molecular Network, West China Hospital, Sichuan University, Chengdu, Sichuan, China; ^7^ School of Pharmaceutical Sciences, Lovely Professional University, Phagwara, Punjab, India

**Keywords:** neuropathic pain, nociception, Parkinson’s disease, Alzheimer’s disease, depression, medicinal plants, polyphenols

Neurological disorders (ND) are now globally recognized as the primary cause of death and disability. The prevalence, incidence, deaths, and disability-adjusted life years (DALY’s) were estimated from 1990 to 2016 in 195 countries for nearly 15 common neurological disorders (Feigin et al., 2019). Based on this detailed data, neurological conditions like stroke, migraine, Alzheimer’s and dementias, and meningitis were the leading cause of DALY’s (276 million) and the second leading cause of death (9 million). The prevalence and incidence of neurological conditions worldwide is alarming (Pringsheim et al., 2014). The prevalence of dementia, epilepsy, Parkinson’s disease, and Huntington’s disease is 4,628, 596, 315, and 2.71 per 1,00,000 persons, respectively, whereas the incidence rate is 4,169, 51.32, 102, and 0.38 per 1,00,000 persons, respectively. It has been reported that about 20 million Americans experience some form of neuropathy in a year (Pal, 2018). About 16% of households in the US contain an individual with brain impairment. Annually, the total number of epilepsy episodes recorded in America is 35 million. It was estimated in 2015 that more than 300 million people globally were suffering from anxiety and depression (WHO, 2017a). Depression is ranked first as a global disability, and anxiety disorders are ranked sixth as per WHO. It is estimated that the global population suffering from depression and anxiety is 4.4% and 3.6%, respectively.

The rapid development of India resulted in an increased burden of neurological disorders, which form a significant proportion of non-communicable diseases. A survey on the burden of neurological disorders in India revealed that non-communicable neurological disorders doubled, whereas communicable neurological disorders reduced to about one-fourth from 1990 to 2019 (Mehndiratta and Aggarwal, 2021). The prevalence rate of common neurological disorders in the urban population of India is higher than in the rural population, with an average of 2,394 per 1,00,000 persons (Gourie-Devi, 2018). The spectrum of neurological disorders in India is similar to what is observed worldwide. The annual incidence rate of strokes is higher, followed by dementia, Parkinson’s disease, and epilepsy. A study performed by NHMS in 2015–16 reported that one in 20 people over the age of 18 have suffered from depression in India (WHO, 2017b). This figure amounts to more than 45 million persons with depression in India. Mental, neurological, and substance use disorders account for 31 million DALYs. Of these, 37% (11.5 million) DALYs are due to depression.

In the present era, a significant increase in neurological disorders has been observed throughout the globe due to the COVID-19 pandemic or as co-morbidities of post COVID-19 complications. The partial or complete lockdown situations in the last 2 years have confined everyone in their respective territories, leading to changes in neurological complications like anxiety, depression, stress, and psychological disturbances in the general public, especially in children. It has been reported that isolation and post-traumatic stress was the leading cause of depression and altered mood in post COVID-19 patients (Rogers et al., 2020). A study showed that 78 out of 214 patients with COVID-19 developed neurological complications such as headache, dizziness and impaired consciousness (Wang et al., 2020). The synthetic drugs and pharmacological interventions currently available for treating neurological disorders are associated with severe adverse effects. Managing such disorders with natural products seems to be a viable approach.

It is the hour of need to deal with the increasing number of neurological disorders in developing and developed countries with safer and therapeutically efficacious medicines. We can achieve this goal with advanced clinical and translational research and bioinformatics tools on traditionally used and medicinally potential natural products (herbal drugs). Considering the need to enrich more information about natural products and neurological disorders, researchers and clinicians working in the Natural Products/Pharmacology field were invited to contribute their valuable research to this Research Topic article collection. Research articles on pre-clinical and clinical studies of Natural Products/Traditional Plants in neurological disorders (Anxiety, stress, Depression, Epilepsy, Dementia, Alzheimer, etc.), Case studies/clinical reports emphasizing the role of natural products in neurological disorders, and Review articles focusing on “Neuropharmacological Studies of Natural Products” covering Clinical and translational research with bioinformatics tools, SAR and molecular docking studies and Mechanistic Insights were considered under the scope of this Research Topic.

We received ten articles for consideration for publication in the thematic issue. After a thorough review, five articles were accepted out of 10 submitted articles. Amongst these, two articles are original research papers, one systematic review, one review, and one mini-review. A review article published by Puri et al., entitled “Natural product-based pharmacological studies for neurological disorders,” focused on targets associated with neurological disorders, traditional holistic approaches for managing neurological disorders and mechanistic approach of phytoconstituents in treating various neurological disorders. A mini-review entitled “Understanding the role of “sunshine vitamin D” in Parkinson’s disease: A review,” published by Behl et al., explained the role of sunshine vitamin D in Parkinson’s disease. In the systematic review article entitled “The neuroprotective effects of melatonin against diabetic neuropathy: A systematic review of non-clinical studies,” published by Hosseini et al., the authors have critically analyzed the role of melatonin in diabetic neuropathy and explained its mechanisms taking reference from pre-clinical data. A research paper entitled “Evaluation of Mollugo oppositifolia Linn. as cholinesterase and *β*-Secretase enzymes inhibitor,” published by Das et al., emphasized *in vitro* neuroprotective effect of *Mollugo oppositifolia* in Alzheimer’s disease. The authors have identified phytochemicals in bioactive plant extract and performed *in silico* molecular docking studies to identify more potential and stable metabolites with potential for Alzheimer’s disease targeting. Another research paper, “Long-term oral administration of naringenin counteracts aging-related retinal degeneration via regulation of mitochondrial dynamics and autophagy,” stressed the role of a CNS-acting flavonoid, Naringenin, in counteracting aging-related retinal degeneration.
